# Selected Chemokines as Prognostic Biomarkers and Therapeutic Targets in Ovarian Cancer

**DOI:** 10.3390/cimb48070673

**Published:** 2026-06-30

**Authors:** Anna Długaszek, Jacek Kabut, Małgorzata Domagała-Haduch, Anita Gorzelak-Magiera, Joanna Sadurska, Maria-Laura Morawiec, Aleksandra Mielczarek-Palacz, Iwona Gisterek-Grocholska

**Affiliations:** 1 Department of Oncology and Radiotherapy, Medical University of Silesia, 40-055 Katowice, Poland; 2 Clinical Oncology Unit, The Professor K. Gibiński University Clinical Center of the Silesian Medical, Medical University of Silesia, 40-055 Katowice, Poland; 3 Department of Immunology and Serology, Faculty of Pharmaceutical Sciences in Sosnowiec, Medical University of Silesia, 40-055 Katowice, Poland

**Keywords:** ovarian cancer, chemokines, tumor microenvironment, chemoresistance

## Abstract

Ovarian cancer, particularly high-grade serous ovarian cancer (HGSOC), remains one of the most lethal gynecological malignancies due to late diagnosis and the development of chemoresistance. The tumor microenvironment (TME) plays an important role in disease progression, with chemokines influencing cell recruitment, angiogenesis, metastasis, and immune modification. This review synthesizes current evidence on key chemokine axes in ovarian cancer, highlighting their dual roles as prognostic biomarkers and therapeutic targets. The most important axes include CXCL12/CXCR4 (which drives tumor proliferation, angiogenesis and chemoresistance via epithelial–mesenchymal transition), CCL2/CCR2 (promoting immunosuppressive tumor-associated macrophages and resistance), and CCL5/CCR5 (enhancing pro-oncogenic signaling and Treg/MDSC infiltration). Pro-angiogenic ELR+CXC chemokines like CXCL8 induce vascularization and inflammation. On the contrary, effector chemokines (CXCL9/10/11/13) correlate with “hot” immune subtypes and improved survival in several studies. High expression of immunosuppressive chemokines predicts poorer prognosis and therapy resistance, while immune-attracting profiles associate with better outcomes and chemotherapy responsiveness. Therapeutically, inhibitors like plerixafor (CXCR4), PF-04136309 (CCR2), and maraviroc (CCR5) show preclinical promise, synergizing with chemotherapy, anti-VEGF, and checkpoint inhibitors. Chemokines also represent actionable molecular targets to overcome ovarian cancer’s “cold” immune phenotype. Future research should validate multi-chemokine signatures for patient stratification and advanced clinical trials toward personalized therapies.

## 1. Introduction

Ovarian cancer is one of the most lethal gynecological cancers [1]. Due to its late diagnosis, most patients are diagnosed at an advanced stage (FIGO stage III–IV). Despite significant advances in treatment in recent years, fewer than half of patients survive 5 years after diagnosis, and ovarian cancer is still considered a chronic disease with alternating periods of remission and relapse [2]. The predominant histological type is high-grade serous ovarian cancer (HGSOC), which is characterized by an aggressive course and a high tendency to recur. Standard therapy is cytoreductive surgery and platinum-based chemotherapy [3]. Depending on the molecular profile of the tumor and the response to treatment, therapy is supplemented with bevacizumab and/or PARP (ADP-ribose polymerase) inhibitors. One of the priorities remains the search for new therapeutic targets, biomarkers of treatment resistance, and attempts to personalize therapy.

In recent years, researchers have focused on the tumor microenvironment and the role of the immune system in inhibiting or promoting ovarian cancer progression. Data have also emerged indicating significant contribution to the development of chemoresistance [4]. Chemokines are a family of low-molecular-weight cytokine proteins with chemotactic properties—they play a key role in the recruitment and distribution of immune cells within tumors [5]. By interacting with their receptors on the surfaces of immune cells and tumor cells, chemokines orchestrate the migration of these cells and their interactions, which influences the course of the antitumor response or promotes tumor cell immune escape. Increasing evidence indicates that chemokine expression profiles correlate with tumor aggressiveness and patient prognosis, and may also determine treatment efficacy [6]. Understanding the function of chemokines in tumor biology may therefore lead to the development of new biomarkers and therapeutic strategies to modify the immune response in patients with advanced ovarian cancer. This review summarizes current evidence on key chemokine axes in ovarian cancer and discusses their potential as prognostic biomarkers and therapeutic targets.

## 2. Chemokines in the Ovarian Tumor Microenvironment Have Potential as Therapeutic Targets

The tumor microenvironment (TME) of ovarian cancer consists of tumor cells, infiltrating leukocytes (including macrophages, lymphocytes, and neutrophils), cancer-associated fibroblasts (CAFs), endothelial cells, and a number of other factors. Chemokines produced by tumor and stromal cells are responsible for the recruitment of specific leukocyte populations to the tumor environment and their distribution within the tissue [7]. In “immunologically cold” cancers, which classically include ovarian cancer [8], impaired antigen presentation, lack of effector T cell infiltration, activation of immune escape pathways, and increased infiltration by immunosuppressive cells (e.g., regulatory T cells—Treg) are common [9]. Chemokines can influence the described phenomena in two ways: by acting immunosuppressively (worsening prognosis) or by promoting an antitumor response (improving prognosis).

The growing knowledge of the role of chemokines in the development of cancer and modulating local and systemic immune responses has led to attempts to utilize their pathways as therapeutic targets. Therapeutic strategies may involve blocking chemokine receptors, neutralizing the chemokine ligands themselves, or disrupting chemokine-receptor signaling. There are currently many studies underway at the clinical or preclinical stage that focus on the CXCL12/CXCR4, CCL2/CCR2, and CCL5/CCR5 axes, which are best known for their role in the development of ovarian cancer. It is important to distinguish between mechanistic rationale, preclinical findings, and clinically validated benefit. While many chemokine-targeting strategies demonstrate promising biological effects in experimental models, only a limited number have shown meaningful clinical efficacy to date. Therefore, caution is warranted when interpreting translational potential.

In this review, the focus is placed on chemokine axes that are most consistently implicated in ovarian cancer biology, namely CXCL12/CXCR4, CCL2/CCR2, CCL5/CCR5, ELR^+^ CXC chemokines, and Treg-recruiting chemokines such as CCL22, CCL17, and CCL28. These pathways have been repeatedly associated with key features of advanced ovarian cancer, including peritoneal dissemination, angiogenesis, accumulation of tumor-associated macrophages and regulatory T cells, platinum resistance, and the modulation of response to immunotherapy. Although these chemokines are also involved in other solid tumors, their combined contribution to the “cold” immune phenotype and extensive peritoneal spread makes them particularly relevant in the context of high-grade serous ovarian cancer.

For clarity, each chemokine axis is discussed using a standardized framework that includes biological function, signaling mechanisms, clinical relevance, therapeutic targeting strategies, and current limitations.

## 3. The CXCL12/CXCR4 (SDF-1/CXCR4) Axis

**Figure 1:**
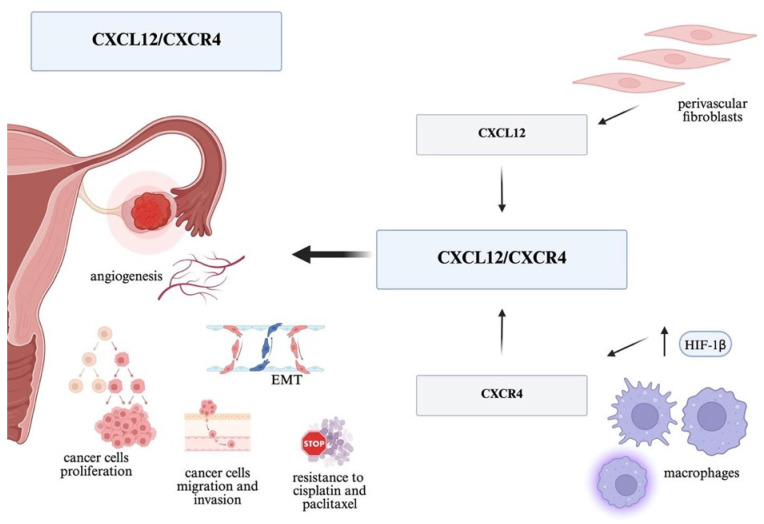
The role of the CXCL12/CXCR4 axis in ovarian cancer [10,11,12,13,14,15,16].

CXCL12 and its canonical receptor CXCR4 constitute one of the best-characterized chemokine axes in oncology. Under physiological conditions, this pathway regulates stem cell homing, organ development, and immune cell trafficking, whereas in cancer, it is frequently hijacked to promote tumor growth and metastasis across multiple tumor types. This is also the best-documented axis associated with the aggressive course of ovarian cancer (Figure 1). The chemokine CXCL12 (also known as SDF-1) plays a significant role in tumor dissemination. Studies in a mouse model have shown that overexpression of CXCR4 in SKOV3 cells (a human ovarian cancer cell line) enhances their proliferation, migration, invasion, and formation of implants in the peritoneal cavity in response to CXCL12 both in vitro and in vivo [10]. Hypoxia increases HIF1α levels and leads to a significant increase in CXCR4 expression and enhanced migration and invasion of tumor cells, further increasing their propensity for metastasis [11]. The CXCL12/CXCR4 pathway also participates in tumor angiogenesis—CXCL12 is a known proangiogenic factor that facilitates the formation of new blood vessels within the tumor mass. In cell model studies, the level of vascular endothelial growth factor (VEGF) mRNA correlates directly with the level of CXCR4 mRNA in tumor cells. CXCL12/CXCR4 signaling activates several downstream pathways, including PI3K/AKT, MAPK/ERK, and JAK–STAT, which together promote tumor cell survival, proliferation, migration, and resistance to apoptosis [12]. Furthermore, model studies have shown that the cytokine TGF-β secreted by cancer cells can induce the increased expression of CXCR4 in nearby macrophages—these then accumulate along the CXCL12 gradient near perivascular fibroblasts and facilitate tumor cell entry into the circulation [13]. Several studies indicate that high CXCL12 expression is an independent predictor of shorter overall survival (OS) and progression-free survival (PFS). Popple et al. assessed CXCL12 and CXCR4 expression using immunohistochemistry in 289 ovarian cancer tissues and demonstrated that high levels of CXCL12 (but not CXCR4) significantly shorten disease-specific survival, regardless of FIGO stage, residual disease after surgery, and prior adjuvant chemotherapy [14]. This axis also promotes chemotherapy resistance by inducing epithelial–mesenchymal transition (EMT). In ovarian cancer models, CXCL12/CXCR4 stimulation has been shown to enhance EMT, which is associated with resistance to cisplatin and paclitaxel, among others, while CXCR4 blockade reverses EMT and restores cell sensitivity to chemotherapy [15,16]. CXCR7 also functions as a receptor for SDF-1 and plays a significant role in cancer development; it enhances cell invasion via matrix metalloproteinase (MMP)-9 and may represent a potential therapeutic target [17].

## 4. The CXCL12/CXCR4 Axis Inhibitors

The CXCR4 receptor has long been an attractive therapeutic target—not only because of its role in the mechanism of cancer metastasis, but also because of its importance in the cancer cell environment (Figure 1). A drug targeting CXCR4 used in hematology is plerixafor (AMD3100), used, among other things, to mobilize stem cells prior to bone marrow transplantation. It is a bicyclam derivative and a reversible CXCR4 receptor antagonist [18]. In preclinical models, its blockade has demonstrated promising antitumor effects: it inhibits tumor cell migration to other organs and can “expose” the tumor to the immune system. For example, inhibition of CXCR4 in a mouse model of ovarian cancer inhibited intraperitoneal dissemination and tumor growth, reduced metastasis, and significantly prolonged mouse survival, including by enhancing CD8+ T cell responses and reducing immunosuppression in the TME [19]. An interesting idea is to attempt combination therapy—in experimental models, the AMD3100 inhibitor (plerixafor) synergized with an anti-PD-1 antibody, leading to significant regression of pancreatic tumors [20]. Zeng et al., in their 2019 study, used a similar combination in a mouse model of ovarian cancer, achieving the inhibition of tumor growth, limiting the spread of tumor cells, increasing the infiltration of effector T lymphocytes, and reducing the percentage of Tregs, which translated into longer survival [21]. Another CXCR4 inhibitor, BL-8040 (motixafortide), combined with anti-PD-1 immunotherapy, also yielded encouraging results in a clinical trial in patients with immunotherapy-resistant pancreatic cancer. The COMBAT study, a single-arm phase II study that added motixafortide to pembrolizumab and chemotherapy in patients with metastatic pancreatic cancer after gemcitabine treatment, yielded promising disease control rates (DCR) of 63% and objective response rates (ORR) of 13–21%, and demonstrated TME modification (increase in activated CD8+ T cells and decrease in MDSC). These results suggest that CXCR4 blockade may overcome resistance to immunotherapy by transforming the “cold” TME into an environment more conducive to T cell activity. In the context of ovarian cancer, research is underway to use CXCR4 antagonists to inhibit the intraperitoneal dissemination of cancer cells. Although there are no reports of large clinical trials of CXCR4 inhibitors in ovarian cancer, their potential is supported by in vitro studies—for example, inhibition of SDF-1/CXCR4 signaling reduced ovarian cancer cell invasion stimulated by stress factors [22]. Research is also underway on radiopharmaceuticals targeting the CXCR4 receptor, which could enable precise drug delivery to microfoci of tumor implants, particularly in the peritoneum [23]. In summary, CXCR4 inhibitors (such as plerixafor and newer molecules) represent a promising class of drugs that may limit ovarian cancer metastasis and improve the efficacy of immunotherapy by modulating the tumor microenvironment.

## 5. The CCL2(MCP-1)/CCR2 Axis

**Figure 2:**
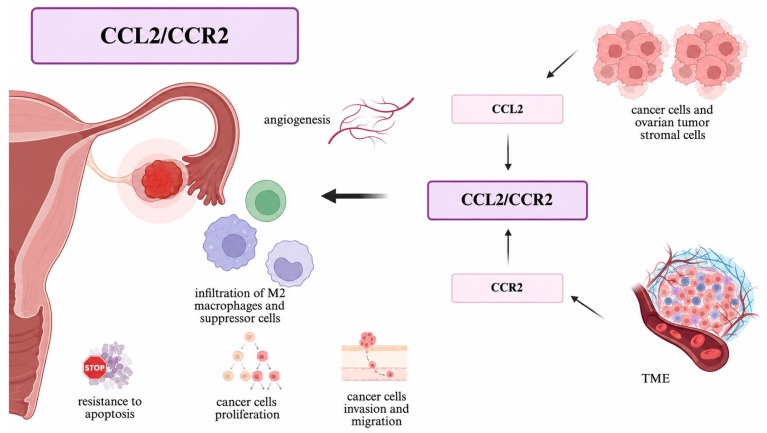
The role of the CCL2/CCR2 axis in ovarian cancer [24,25].

The CCL2 chemokine (also known as MCP-1) is primarily responsible for the recruitment of monocytes and macrophages to the tumor via CCR2 receptors. Ovarian cancers contain a high percentage of tumor-associated macrophages (TAMs), particularly those of the M2 phenotype. These are considered immunosuppressive. They act pro-tumorigenically in the TME, supporting growth and angiogenesis, suppressing the immune response, and promoting metastasis. CCL2, secreted by tumor cells and ovarian tumor stromal cells, attracts monocytic precursors to the TME, which differentiate into immunosuppressive TAMs, resulting in a microenvironment conducive to immune escape. Importantly, CCL2/CCR2 signaling not only increases the infiltration of M2 macrophages and other suppressor cells (including regulatory T cells and myeloid suppressor cells—MDSCs), but also directly stimulates the cancer cells themselves, promoting their proliferation, invasiveness, migration, and resistance to apoptosis. As a result, the CCL2/CCR2 axis contributes to metastasis, angiogenesis, and treatment resistance [24,25] (Figure 2).

## 6. Therapies Blocking CCL2/CCR2 Route

Inhibition of the CCL2/CCR2 axis aims to limit the influx of M2 macrophages and other suppressor cells into the tumor, thereby unblocking immune suppression. In animal models of many cancers, blocking this pathway has yielded encouraging results—inhibition of CCL2 signaling (e.g., with neutralizing antibodies or chemokine traps) resulted in a reduction in the number of TAMs and tumor cell migration, which translated into slower tumor growth and reduced metastasis (Figure 2). At the molecular level, CCL2/CCR2 signaling in tumor and myeloid cells engages the JAK–STAT and NF-κB pathways, driving the expression of proangiogenic and immunosuppressive mediators and reinforcing the tumor-supportive phenotype of tumor-associated macrophages [26,27]. Similarly, CCR2 antagonist molecules (such as RS-504393 or RS-102895) inhibited the infiltration of immunosuppressive macrophages, delaying tumor progression [28]. In recent years, these approaches have begun to be translated into clinical trials. The monoclonal antibody carlumab (anti-CCL2) was tested in a phase I trial in patients with advanced solid tumors. The drug was well-tolerated, although the antitumor effect in monotherapy was limited (stable disease in some patients). More promising results were achieved with the combination of the small-molecule CCR2 inhibitor PF-04136309 with FOLFIRINOX chemotherapy in patients with pancreatic cancer. In a phase Ib trial, a 97% disease control rate was achieved and an objective response rate of 49% was achieved. These data suggest that CCR2 blockade can significantly increase the efficacy of chemotherapy, likely by limiting TAM infiltration and sensitizing tumor cells to cytotoxic drugs [29]. Based on this, trials combining CCR2 inhibitors with chemotherapy are planned or underway in other cancers, including potentially ovarian cancer. It is worth noting that combining CCL2/CCR2 blockade with immunotherapy demonstrates a synergistic effect: in preclinical models, simultaneous inhibition of TAM influx (with a CCR2 inhibitor) and the administration of an anti-PD-1 antibody significantly inhibited the growth of tumors previously resistant to immunotherapy. Li X et al. demonstrated in several mouse models of solid tumors that pharmacological blockade of CCR2 or genetic silencing of CCR2 reduces TAM influx, and combining this inhibition with an anti-PD-1 antibody significantly inhibits the growth of tumors resistant to monotherapy, increases CD8+ infiltration, and significantly prolongs animal survival [30]. This bidirectional approach—simultaneous silencing of immunosuppression by CCR2 and activation of T lymphocytes by checkpoint inhibitors—is an attractive therapeutic option for “immunologically cold” tumors such as ovarian cancer and may become the subject of further clinical trials. In 2022, Zhai et al. published a study in which the combination of a CCR2 inhibitor with bevacizumab reduced M2TAM infiltration, inhibited tumor vascularization and growth, and overcame anti-VEGF resistance, particularly in the serous subtype of ovarian cancer [31].

The expression of B7-H3 (CD276) in tumor cells contributes to CCL2–CCR2–M2 macrophage axis-mediated immunosuppression and tumor progression. B7-H3 is expressed in non-immunoreactive HGSOC tumors with low PD-L1 levels, and its expression was negatively correlated with the IFNγ signature, which reflects the tumor’s immunological reactivity. In mouse models lacking the B7-H3 gene, tumor progression was inhibited, with a reduced number of M2 macrophages and an increased number of IFNγ+CD8+ T cells. Inhibition of the CCL2–CCR2 axis partially counteracted the effect of B7-H3 suppression on the migration and differentiation of pro-tumor M2 macrophages and tumor progression. In patients with HGSOC, B7-H3 expression positively correlated with CCL2 expression and the number of M2 macrophages. Patients with tumors exhibiting high B7-H3 levels had fewer IFNγ+CD8+ T cells in the tumor and had a poorer prognosis than patients with tumors exhibiting low B7-H3 levels [32].

In summary, targeting the CCL2/CCR2 axis is a promising therapeutic strategy aimed at reprogramming the ovarian tumor microenvironment by reducing the number of TAMs and other cells that promote tumor growth. Further studies are needed to determine the safety and duration of such therapies.

## 7. The CCL5 (RANTES)/CCR5 Axis

**Figure 3:**
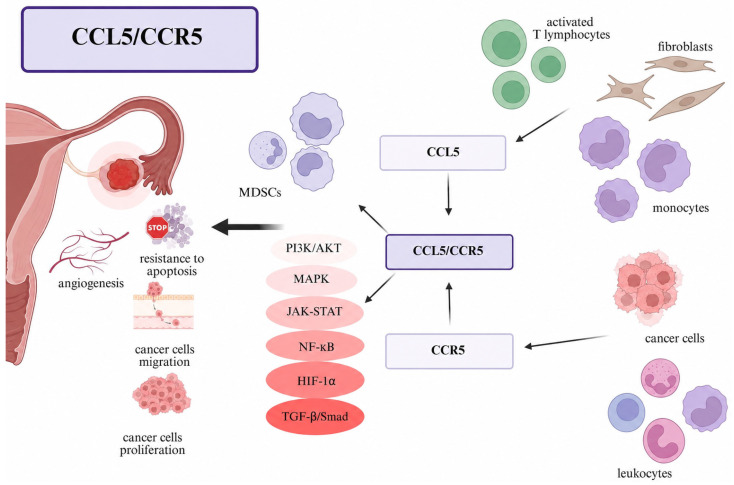
The role of the CCL5/CCR5 axis in ovarian cancer [33,34,35].

CCL5 is a chemokine produced by activated T lymphocytes, monocytes, and fibroblasts, which exerts its biological effects through binding to the CCR5 receptor. Signaling through CCL5/CCR5 has a multifaceted function: on the one hand, it recruits regulatory T cells and MDSCs to the tumor, increasing immunosuppression, and on the other hand, it directly activates a number of pro-oncogenic pathways in cancer cells (PI3K/AKT, MAPK, JAK-STAT, NF-κB, HIF-1α, TGF-β/Smad). Stimulation of these pathways results in increased cancer cell proliferation, their ability to migrate and invade, resistance to apoptosis, and increased angiogenesis [33]. CCL5/CCR5 therefore plays a dual role: it promotes the influx of pro-tumor immune cells (Tregs, MDSCs) and directly supports the survival and spread of tumor cells. High CCL5 concentrations in the tumor have been shown to correlate with increased infiltration of immunosuppressive monocytes and Tregs, which accelerates cancer development. The presence of Treg lymphocyte infiltration has been confirmed as a negative prognostic factor in many cancers, including breast, kidney, cervical, and colon cancer [34,35] (Figure 3).

## 8. Blocking CCL5/CCR5 Axis

The CCR5 receptor is primarily known for its role in HIV infection, but it has also emerged as an interesting target in oncology. In ovarian cancer, the CCL5/CCR5 axis may be activated (e.g., peritumoral fibroblasts may secrete CCL5, influencing cancer cells and immune cells), making it a potential treatment target [36]. One of the first CCL-5 antagonists studied was anibamine and its analogues, which, in studies using a cell model of ovarian cancer, significantly inhibited CCL5-induced calcium ion influx in OVCAR3 cells, confirming the blockade of CCL5/CCR5 signaling [37]. A more advanced drug, for example, is maraviroc, long used in the treatment of HIV. Maraviroc has been preclinically tested in various cancer models—its addition to therapy has been shown to reduce metastatic potential [38]. There are also reports that CCR5 blockade may enhance the effectiveness of chemotherapy and immunotherapy. In glioma cell lines, CCL5 produced by perivascular cells has been shown to facilitate DNA repair in tumor cells following temozolomide treatment. Blocking the CCL5–CCR5 interaction with maraviroc abolished this effect—impairing DNA repair, increasing the sensitivity of glioma cells to chemotherapy and resulting in tumor shrinkage in treated mice [39]. Interesting data suggesting an impact on immunotherapy are available: for example, in a melanoma model, an anti-CCR5 antibody reduced the accumulation of myeloid suppressor cells in the tumor, thus enabling the action of anti-PD-1 therapy, leading to the inhibition of tumor growth [40]. This demonstrates that anti-CCR5 therapy may be a valuable complement to other methods. Early-phase clinical trials are currently underway to assess the safety and preliminary efficacy of maraviroc in cancer patients (including advanced colorectal cancer and breast cancer). The PICASSO study was a phase I trial evaluating the combination of pembrolizumab and maraviroc in metastatic colorectal cancer with high microsatellite instability, resulting in acceptable toxicity and cases of stabilization and partial response [41]. In ovarian cancer, this treatment should be considered, particularly in patients with high CCL5/CCR5 expression in the tumor microenvironment, in combination with standard therapy. In summary, blocking the CCL5/CCR5 axis (e.g., with maraviroc) appears to be an interesting strategy for increasing tumor sensitivity to other therapies, although this remains to be confirmed in larger clinical trials.

## 9. CXCL8 and Others from ELR^+^ CXC Family

The family of so-called ELR-positive chemokines includes interleukin 8 (CXCL8), characterized by the amino acid sequence Glu-Leu-Arg (ELR) preceding the classic Cys-X-Cys (CXC) [42]. Chemokines from this group have potent proangiogenic properties, and CXCL8 is present in high concentrations in peritoneal fluid and ovarian tumor tissue. It binds to CXCR1/2 receptors on neutrophils, macrophages, endothelial cells, and the tumor cells themselves. Activation of CXCR1/2 triggers a signaling cascade (including the PI3K/AKT, PLC/PKC, and JAK-STAT pathways), resulting in a strong proinflammatory and proangiogenic effect. Under normal conditions, CXCL8 stimulates angiogenesis and healing, but in cancers, it leads to abnormal tumor vascularization, cell growth, and facilitated tumor migration. Excessive activation of the CXCL8–CXCR1/2 axis has been documented in ovarian cancer, where it contributes to increased tumor cell proliferation and neoangiogenesis. In addition to CXCL8, related chemokines with the ELR motif (e.g., CXCL1-3) may also have similar proangiogenic and proinvasive functions in ovarian cancer [43,44]. Moreover, the CXCL8-CXCR2 axis promotes the polarization of pro-tumor M2 macrophages via RASGRP4-dependent mTOR-STAT3 signaling in ovarian cancer. Downregulation of the axis components and RASGRP4 reduced tumor growth and M2 macrophage infiltration [45].

## 10. Chemokines Recruiting Regulatory T Cells

Ovarian cancers also utilize certain chemokines to actively attract immune-suppressing cells to the tumor. CCL22 (produced primarily by TAMs) and CCL17 have been shown to interact with the CCR4 receptor present on Treg cells, facilitating their recruitment [46]. Ovarian cancer is characterized by the high infiltration of tumor tissue by highly immunosuppressive Treg lymphocytes expressing the FOXP3+ protein, and their number correlates with poorer prognosis and suppression of the antitumor response [47]. Similarly, the chemokine CCL28, induced under hypoxic tumor cell conditions, attracts another pool of Treg lymphocytes via the CCR10 receptor to the ovarian cancer microenvironment. The influx of these cells contributes to the immunologically “cold” tumor phenotype, in which cytotoxic T and NK lymphocytes are scarce and the potential response to immunotherapy is poor [48,49]. In summary, chemokines such as CCL22, CCL17, and CCL28 are additional factors that support the creation of an immunosuppressive TME and promote immune escape.

## 11. Other Approaches and Combined Therapies

In addition to the above-mentioned interventions, a number of other interventions targeting the chemokine system are under development. For example, CXCR2 receptor inhibitors are in the preclinical phase; their goal is to limit neutrophil influx and inhibit angiogenesis. In an ovarian cancer model, the CXCR2 inhibitor SB225002 was shown to inhibit the action of ELR^+^ chemokines (CXCL1/2/3/5/8), reduce cell proliferation, limit angiogenesis, and—in combination with sorafenib—prolong the antiangiogenic effect and delay the development of resistance to anti-VEGF therapy [50]. Concurrently, approaches to neutralizing Treg chemokines—CCL22/CCL17—are being tested, for example, through “decoy antibodies” [51] or the administration of an anti-CCR4 antibody (mogamulizumab), which eliminates circulating and tissue Tregs [52]. Mogamulizumab is already approved for cutaneous lymphomas, and studies in solid tumor models (including ovarian) suggest that it may reduce the percentage of Tregs in the microenvironment and enhance the antitumor response [53]. Another solution is the use of so-called chemokine fusion—scientists are testing, for example, combining tumor-targeting antibody fragments with chemokine components, which is intended to direct T lymphocytes directly to tumor cells [54]. This approach—although still in the early phase—may in the future increase the precision of immunotherapy by directing effector cells to where they are most needed. Combining anti-chemokine drugs with other therapies is also an extremely interesting approach: the described examples (CCR2 + PD-1; CCR5 + PD-1; CXCR4 + PD-1; CCR2 + chemotherapy, etc.) show that these combinations produce a synergistic effect. This is because blockade of, for example, one chemokine receptor alone can be compensated by other tumor mechanisms, but combining it with an attack on a key growth pathway or immune checkpoint can contribute to the breakdown of tumor defenses. Early-phase clinical trials are currently underway on combination therapies, such as a CSF-1R inhibitor (which eliminates macrophages) with a CXCR4 inhibitor or checkpoint inhibitors, which are intended to reverse immunosuppression in the tumor environment [55,56].

Another potential therapeutic target could be the CCL25/CCR9 axis, whose components have been observed to be highly expressed across multiple ovarian tumor subtypes, including serous, endometrioid, mucinous, clear cell and sex cord–stromal tumors, compared with non-neoplastic ovarian tissue [57,58].

Similarly, the CCL20/CCR6 axis promotes ovarian cancer metastasis both in vivo and in vitro, likely by increasing tumor cell adhesion and the epithelial–mesenchymal transition [59]. Also, the inhibition of CCL20 or CCR6 inhibits ovarian cancer migration induced by cisplatin-stimulated classically activated macrophages (Cis-CAMs) [60,61]. The CXCL16/CXCR6 axis influences the migration and invasion of ovarian cancer cells via macrophages, and silencing CXCR6 impairs this ability, which also makes this axis a therapeutic target. The CCL19/CCR7 axis is also involved in EMT during the development of EOC; however, the potential therapeutic role of the axis itself has not been investigated [62].

## 12. Chemokines as Prognostic and Predictive Biomarkers

The chemokine expression profile in ovarian cancer provides valuable information about the biological characteristics of the tumor and may have prognostic and predictive significance (predicting response to therapy). Both clinical and translational studies in recent years have indicated a number of correlations between the concentration of specific chemokines or their receptors and the course of the disease in patients with ovarian cancer.

## 13. A “Hot” Immune Subtype with Effector Chemokine Expression

Zhuo et al. performed a transcriptomic analysis of HGSOC and defined an immunologically “hot” subtype with high expression of four chemokines—CXCL9, CXCL10, CXCL11, and CXCL13—which comprised approximately 20–30% of cases and was characterized by increased infiltration of effector lymphocytes and a better prognosis [63]. These chemokines are responsible for the increased influx of T lymphocytes and NK cells into the tumor (CXCL9–11 ligands activate the CXCR3 receptor on cytotoxic CD8+ T lymphocytes and NK cells, while CXCL13 attracts B lymphocytes via CXCR5). Patients with tumors exhibiting these characteristics had longer overall survival and longer relapse-free survival (RFS) compared to patients with low levels of these chemokines. Importantly, this four-chemokine prognostic signature was confirmed in independent multicenter cohorts as a favorable prognostic factor, highlighting the potential of CXCL9/10/11/13 as biomarkers of good prognosis. These results are consistent with previous observations that the presence of infiltrates composed of activated Th1 and Tc lymphocytes (marked, among others, by CXCL9–11 expression) is associated with longer survival in patients with ovarian cancer. Bronger et al. [64] published a study in 2016 demonstrating that high tumor CXCL9 and CXCL10 concentrations correlate with greater CD3+ T cell infiltration and approximately twofold longer overall survival in HGSOC.

## 14. High Levels of Immunosuppressive Chemokines–Poorer Prognosis

Overexpression of chemokines associated with chronic inflammation and immunosuppression is associated with a less favorable disease course. Lane et al. demonstrated that elevated IL8 levels in peritoneal fluid coexist with advanced clinical stage and greater angiogenic activity, and correlate with a shorter progression-free survival [65]. Studies have shown that patients with elevated CXCL8 levels in the blood have a significantly poorer prognosis. A study performed in 2013, analyzing 184 patients, noted that high serum IL8 and IL-6 levels were independently associated with increased mortality [66]. In mouse models, overexpression of IL8 in ovarian tumor tissue is associated with a higher stage and poorer clinical outcome, while reduced IL8 levels inhibit tumor growth through antiangiogenic effects [67]. High CCL2 expression has also been associated with an aggressive tumor phenotype and chemotherapy resistance. In the study by Moisan et al., blocking CCL2 in a mouse model was associated with a better response to standard chemotherapy based on paclitaxel and carboplatin [68]. CCL2 is responsible, among other things, for increased TAM infiltration within the tumor, which is a proven factor for poor prognosis [24]. Interestingly, one immunohistochemical study demonstrated that a high density of CXCL12-positive immune cell infiltration (i.e., the presence of many SDF-1-secreting cells in the tumor) was paradoxically associated with a better response to platinum-based chemotherapy and a longer relapse-free survival. The same study found that patients whose primary tumors had a higher expression of CXCL12+ immune cells were more likely to achieve a response to first-line chemotherapy and had a lower incidence of relapse [69]. The authors suggest that the presence of SDF-1-producing cells (perhaps specific fibroblast subtypes or lymphoid effector cells) promotes the recruitment of effector lymphocytes to the tumor, which improves the efficacy of chemotherapy by improving immunological control of the tumor. CXCL12 is believed to exert a bidirectional, concentration-dependent effect on multiple cell types. Further studies are needed to identify precisely which infiltrating cells express CXCL12 and how they influence the tumor microenvironment during treatment.

## 15. Chemokine Receptor Activation Indicators as Markers of Treatment Response

A new approach is to assess not only the levels of the chemokines themselves, but also the activation status of their receptors in immune cells. For example, in 2022, Walther et al. investigated the importance of active signaling through the CXCR4 receptor on tumor immune infiltrate cells. They assessed the ratio of phosphorylated (active) CXCR4 to total CXCR4 levels in infiltrating leukocytes. A high pCXCR4/CXCR4 ratio in primary tumor immune cells proved to be an independent predictor of longer RFS and a better response to first-line chemotherapy. In other words, patients whose tumors demonstrated strong activation of the CXCR4 pathway in immune cells were less likely to experience early relapse after treatment [70]. This suggests that CXCR4 signaling in certain leukocyte populations (possibly Th1 lymphocytes, NK cells, or dendritic cells mobilized from the blood by SDF-1), in combination with chemotherapy, promotes effective immune cell eradication of tumor cells. In the longer term, the pCXCR4/CXCR4 ratio alone no longer significantly correlated with survival, which may be due to dynamic changes in the TME after subsequent lines of treatment. Nevertheless, this report emphasizes that analysis of the activation status of chemokine receptors in the tumor microenvironment can provide additional predictive information and should be further developed in future studies.

In the context of the SDF-1/CXCR4 axis, the researchers also directly analyzed the expression of these proteins in tumor tissue and prognosis. Material from 289 patients was analyzed, with a follow-up of 14 years. The results are inconsistent—high expression of the CXCL12 ligand in ovarian cancer cells was associated with a shorter time to relapse, whereas tumor expression of CXCR4 alone had no significant impact on survival [14]. The authors suggested that tumor SDF-1 levels could serve as a prognostic marker—for example, patients with high CXCL12 levels are at higher risk of relapse, which could influence therapeutic decisions. Furthermore, they emphasized that proteins associated with the CXCL12/CXCR4 complex may be potential treatment targets. These observations support the hypothesis that blocking the SDF-1/CXCR4 axis could particularly benefit patients whose cancer strongly utilizes this pathway for progression. The seemingly contradictory observations regarding the importance of the CXCL12/CXCR4 axis could be explained by the different roles of this axis depending on the cell type and the context of the tumor microenvironment. While high CXCL12 production by cancer cells can promote proliferation, angiogenesis, and immunosuppression, activation of the CXCR4 receptor in immune cells may promote their recruitment to the tumor and support an antitumor response, resulting in a better response to chemotherapy and a longer relapse-free survival. It is also noteworthy that resistance to treatment is prevalent in ovarian cancer. The CXCL16/CXCR6 axis plays a role in the resistance of ovarian cancer cells to cisplatin [71]. Increased expression of the CXCL16/CXCR6 axis components is also associated with a more aggressive form of the disease and a higher incidence of metastasis [72,73].

Given the marked heterogeneity of ovarian cancer, it is unlikely that chemokine-targeted therapies will demonstrate uniform efficacy across all patient populations. Identification of predictive biomarkers is therefore critical for effective clinical translation. Potential strategies include chemokine expression profiling, assessment of immune cell infiltration patterns, and spatial analysis of ligand–receptor interactions within the tumor microenvironment. Integration of these parameters into composite biomarker models may enable more accurate patient stratification and improve therapeutic outcomes.

In summary, chemokines represent promising biomarkers in ovarian cancer. High levels of immune-stimulating chemokines are associated with a better prognosis and greater efficacy of immunotherapy, while a predominance of chemokines associated with suppressor cell recruitment (TAMs, Tregs) correlates with a more aggressive course and treatment resistance. However, the use of these markers in clinical practice requires standardized assays and prospective validation—currently, no single chemokine profile is routinely used as a prognostic or predictive test in gynecologic oncology.

## 16. Clinical Perspectives and Future Research Directions

Applying knowledge about chemokines to clinical practice holds great promise, but also challenges. First, the chemokine system is highly complex. Many chemokines have overlapping functions, and cells can express several different receptors—blocking one pathway can therefore be compensated by another. Furthermore, the same receptors can be expressed on cells with opposing functions: for example, CXCR3 is present on both cytotoxic T lymphocytes (anti-tumor) and regulatory Tregs (tumor-promoting); [74] similarly, CCR5 is found on activated effector lymphocytes, but also on monocytes differentiating into TAMs [75]. From a clinical perspective, this means that anti-chemokine therapies can have bidirectional effects, for example, by inhibiting certain harmful populations while simultaneously affecting beneficial ones. For example, blocking CXCR3 would potentially limit the influx of Tregs into the tumor, but would also inhibit the recruitment of Th1 helper lymphocytes into the tumor vessels [76]. Therefore, proper patient selection for specific therapies and combining them to minimize these adverse effects will be crucial. Temporary administration of inhibitors—e.g., short-term blockade just before or during chemotherapy or immunotherapy—may be a solution, but this requires extensive research. Translational validation of chemokine-based biomarkers is essential. As mentioned, numerous studies have demonstrated correlations between chemokine profiles and prognosis and treatment response. However, translating this into practice requires standardization—e.g., establishing threshold expression values, using the best available tests (IHC, ELISA), and confirming that the information from a given biomarker is independent of other factors (stage, BRCA status, degree of resection, etc.). It is possible that in the future, multi-component algorithms will be developed that assess several chemokines simultaneously, for example, creating a tumor chemokine profile that will allow patients to be selected for more or less aggressive treatment. Prospective clinical trials are necessary to confirm the added value of these biomarkers.

Safety and systemic effects should also be considered: chemokines are involved in many physiological processes (e.g., maintaining bone marrow homeostasis, tissue regeneration, and wound healing). Long-term blockade of important pathways (such as CXCR4 or CCR2) can have serious side effects. For example, plerixafor mobilizes blood stem cells into circulation, which is a desirable effect in transplantation, but in cancer patients, it could potentially result in transient leukocytosis. Inhibition of CCR2, in turn, could affect the anti-infective response—macrophages play a role in fighting infections. It will be important to carefully monitor the toxicity of new drugs in clinical trials and seek solutions that minimize risk (e.g., drugs administered for a short period or tumor-targeted therapies with nanocarriers). It is likely that not all ovarian cancer patients will benefit from chemokine-targeted therapies; it is necessary to identify subgroups of patients who will benefit most—for example, those with high TAM/Treg infiltration (for CCR2/CCR5/CCR4 inhibitors) or, conversely, those with “dormant” T lymphocytes in the tumor environment (for CXCR4 inhibitors, to facilitate their access to the tumor). Modern techniques, such as multichannel spatial immunohistochemistry or single-cell sequencing, allow for the creation of spatial maps of the tumor microenvironment [77]. Analysis of such biochemical and spatial patterns of chemokines can help predict the mechanisms responsible for a given tumor’s resistance and how to attack them. This approach requires complex analyses and validation, but it is consistent with the trend of personalized medicine.

Anti-chemokine therapies in the treatment of advanced ovarian cancer are unlikely to replace standard treatment but may complement it. As mentioned, neutralizing pro-tumor chemokines (e.g., TGFβ, VEGF, CCL2, CCL5, CXCL8) can increase the effectiveness of immunotherapy. For example, anti-VEGF antibodies (bevacizumab) are already being successfully used in ovarian cancer in combination with chemotherapy [78]—evidence that modifying the microenvironment provides clinical benefits. The challenge lies in selecting the right combinations and methods—intensive modeling studies are currently underway to facilitate this.

From a translational perspective, targeting chemokine pathways presents significant challenges due to their fundamental role in physiological processes, including immune cell trafficking, hematopoiesis, and tissue homeostasis. Long-term inhibition of receptors such as CXCR4 or CCR2 may lead to unintended systemic effects, including impaired immune surveillance, altered leukocyte distribution, and increased susceptibility to infections. For example, CXCR4 plays a key role in bone marrow niche retention of hematopoietic stem cells, while CCR2 regulates monocyte mobilization from the bone marrow. Disruption of these pathways may therefore lead to unintended systemic effects, such as impaired immune surveillance and increased susceptibility to infections. These considerations highlight the need for careful dose optimization, temporal targeting strategies, and the development of more selective or tumor-restricted therapeutic approaches.

The complexity of chemokine signaling in ovarian cancer requires a systems-level approach that integrates multiple layers of biological data. Advances in single-cell RNA sequencing and spatial transcriptomics have enabled a detailed characterization of chemokine expression patterns and ligand–receptor interactions within the tumor microenvironment. These technologies allow for the identification of spatially organized immune niches, mapping of stromal–immune–tumor interactions, and reconstruction of cell–cell communication networks. Ligand–receptor interactome analyses can reveal dominant signaling pathways responsible for immune suppression or activation in specific tumor regions. Such integrative approaches may facilitate the identification of context-dependent therapeutic vulnerabilities and support the development of more precise and personalized treatment strategies targeting the chemokine network.

One of the limitations of chemokine-targeted therapies is the high degree of redundancy within the chemokine network. Chemokines and their receptors form a complex and highly interconnected system in which multiple ligands can bind to a single receptor, and individual chemokines may activate more than one receptor. As a result, inhibition of a single chemokine axis is often insufficient to produce durable antitumor responses. Tumors can compensate for targeted blockade through the activation of parallel signaling pathways or through dynamic remodeling of the tumor microenvironment. Stromal components such as cancer-associated fibroblasts and tumor-associated macrophages may increase the secretion of alternative chemokines, thereby restoring immune cell recruitment or maintaining immunosuppressive conditions. This adaptive plasticity represents a major obstacle to effective therapeutic targeting. These observations suggest that successful strategies will likely require combination approaches, including simultaneous targeting of multiple chemokine pathways or integration with immunotherapy, chemotherapy, or antiangiogenic treatment. A deeper understanding of network-level interactions between chemokines is therefore essential for the development of effective therapeutic strategies. Current therapeutic strategies targeting chemokine signaling are presented in Table 1.

In summary, chemokines and their receptors represent promising targets in the treatment of ovarian cancer, but their clinical application requires overcoming numerous obstacles. Further translational research is needed to determine which patients and at what stage of the disease will benefit most from such therapies. Multicenter phase III trials will also be necessary to assess whether the addition of chemokine-targeted drugs actually improves patient survival. If these conditions are met, there is a chance that in the coming years, we will see the development of gynecological oncology toward more personalized and more effective treatment of ovarian cancer.

## Figures and Tables

**Table 1:** Therapeutic agents and strategies targeting chemokine pathways relevant to ovarian cancer.

Target Axis/Receptor	Agent/Strategy	Mechanism of Action	Development Stage/Clinical Status	Main Rationale/Key Limitation
CXCL12/CXCR4	plerixafor (AMD3100)	reversible CXCR4 antagonist	approved in hematology; preclinical and early translational oncology	may inhibit dissemination and improve immune infiltration but systemic CXCR4 blockade may affect hematopoiesis
CXCL12/CXCR4	motixafortide (BL-8040)	high-affinity CXCR4 inhibitor	early clinical development in solid tumors	promising tumor microenvironment remodeling but limited ovarian cancer-specific data
CXCL12/CXCR4	CXCR4-targeted radiopharmaceuticals	receptor-directed imaging and drug delivery	preclinical	may improve delivery to peritoneal implants
CCL2/CCR2	carlumab	anti-CCL2 monoclonal antibody	phase I in solid tumors	well tolerated but limited monotherapy activity
CCL2/CCR2	PF-04136309	small-molecule CCR2 inhibitor	early-phase with chemotherapy in pancreatic cancer	improved outcomes but no ovarian data
CCL5/CCR5	maraviroc	CCR5 antagonist	approved for HIV; early oncology trials	repurposing candidate with immunomodulatory potential but limited evidence in ovarian cancer
CCL22/CCL17/CCR4	mogamulizumab	anti-CCR4 monoclonal antibody	approved in hematologic cancers; exploratory in solid tumors	depletes Tregs but may cause broader immune effects
ELR+ CXC/CXCR2	SB225002	CXCR2 inhibitor	preclinical	may reduce angiogenesis and resistance to anti-VEGF therapy

Abbreviations: HIV, human immunodeficiency virus; Treg, regulatory T cell; VEGF, vascular endothelial growth factor.

## Data Availability

No new data were created or analyzed in this study. Data sharing is not applicable to this article.
